# Enhancement of Zika virus infection by antibodies from West Nile virus seropositive individuals with no history of clinical infection

**DOI:** 10.1186/s12865-020-00389-2

**Published:** 2021-01-09

**Authors:** Himanshu Garg, Rose Yeh, Douglas M. Watts, Tugba Mehmetoglu-Gurbuz, Robert Resendes, Bruce Parsons, Fernando Gonzales, Anjali Joshi

**Affiliations:** 1 Center of Emphasis in Infectious Diseases, Department of Molecular and Translational Medicine, Texas Tech University Health Sciences Center, 5001 El Paso Dr, El Paso, TX 79905 USA; 2 Paul L Foster School of Medicine, Texas Tech University Health Sciences Center, El Paso, TX USA; 3 Department of Biological Sciences, University of Texas at El Paso, El Paso, TX USA; 4 Department of Public Health, City of El Paso, El Paso, TX USA

**Keywords:** ZIKV, ADE, WNV, DENV, neutralization, Enhancement, Envelope

## Abstract

**Background:**

Recent outbreaks of Zika Virus (ZIKV) infection and associated microcephaly has raised multiple scientific questions. The close antigenic relatedness between flaviviruses makes diagnosis of specific infection difficult. This relatedness also raises the potential of Antibody Dependent Enhancement (ADE) via cross reactive antibodies to flaviviruses like West Nile Virus (WNV) and Dengue Virus (DENV). Asymptomatic WNV infections are endemic throughout the US creating a large proportion of the population that is seropositive for WNV antibodies. Whether these sero-positive individuals potentially carry ZIKV enhancing antibodies remains unknown.

**Results:**

Serum samples obtained from human subjects with symptomatic or asymptomatic WNV infection from a WNV endemic region in Texas were tested for their ability to enhance or neutralize ZIKV infection. Sero-surveillance data demonstrated a ~ 7% prevalence for WNV antibodies in the population. Sera from both symptomatic and asymptomatic WNV seropositive donors effectively neutralized WNV and to some extent DENV infection. Interestingly, WNV+ sera failed to inhibit ZIKV while significantly enhancing infection. Conversely, ZIKV specific sera effectively neutralized ZIKV, with ADE only evident at lower concentrations. The enhancement of ZIKV via WNV antibody positive sera was likely due to non-neutralizing Envelope (E) antibodies as seen with monoclonal ZIKV E antibodies.

**Conclusions:**

Overall, our findings suggest that WNV antibodies in the sera significantly enhance ZIKV infection in Fc receptor positive cells with limited neutralization activity. Further studies in more relevant models of ADE will be needed to confirm the relevance of these findings in vivo*.*

**Supplementary Information:**

The online version contains supplementary material available at 10.1186/s12865-020-00389-2.

## Background

Zika Virus (ZIKV) is an arbovirus belonging to the family *Flaviviridae* and is predominantly transmitted to man by *Aedes* mosquitoes [1]. Other modes of transmission like nosocomial, perinatal, sexual, transfusion and organ transplantation have also been reported [1]. The virus was identified in 1947 from a rhesus macaque in Uganda during routine Yellow Fever Virus (YFV) surveillance efforts in the Zika forest [2]. Serological evidence of ZIKV specific antibodies has been reported since 1950 in humans in Africa and Asia [3]. Sporadic human cases with mild disease and no reported hospitalizations/deaths resulted in ZIKV being branded as a benign infection despite widespread evidence of virus circulation in sero-surveillance studies [4, 5]. More recently (2007), ZIKV emerged in the Yap Island where ~ 73% of the residents were reportedly infected, but no severe disease manifestations, hospitalizations or deaths were observed [6]. ZIKV reemerged in the Pacific region in 2013 to cause an outbreak in French Polynesia that was associated with neurological disorders, including Guillain-Barré syndrome, myasthenia gravis and microcephaly [7]. Subsequently, the virus spread during 2015 to Brazil where human cases with neurological manifestations were linked with a previous history of viral infection [3]. Thereafter, in 2016, WHO declared association of ZIKV with many cases of microcephaly and neurological disorders designating the infection as a Public Health Emergency of International Concern (PHEIC).

There is strong scientific evidence that the recent ZIKV outbreak was associated with microcephaly and other neurological complications in unborn fetus, collectively referred to as Congenital Zika Syndrome (CZS) [8, 9]. Furthermore, ZIKV infection in adults is associated with Guillain-Barre like autoimmune disorders [10]. Recent studies on tropism and pathogenesis of various ZIKV isolates have identified apoptosis of neuronal progenitor stem cells as a mechanism behind ZIKV pathogenesis [11, 12]. ZIKV pathogenesis is also associated with other factors including changes in the envelope glycosylation [13] as well as changes in non-structural 1 (NS1) [14] and preMembrane (prM) [15] protein that are associated with enhanced transmission of certain isolates.

The recent outbreak of ZIKV and associated CZS was first observed in regions of Brazil where other flaviviruses like DENV are endemic [16]. Keeping in mind the structural similarity between flaviviruses [17], it is tempting to speculate that the phenomenon of Antibody Dependent Enhancement (ADE) may play a role in the occurrence of increased CZS seen in the 2016 ZIKV outbreak. ADE has been extensively studied in DENV infections, where infants born to DENV infected mother’s experienced more severe disease due to the presence of cross reactive/enhancing antibodies [18, 19]. Moreover, the recent outcome of severe DENV disease in seronegative individuals vaccinated with the Dengavaxia vaccine (Sanofi-Pasteur), further supports the role of ADE in exacerbating flaviviral infections [20]. However, the contribution of ADE in pathogenesis of other flaviviruses like WNV and ZIKV remains uncertain suggesting that this phenomenon is more complex than previously thought.

WNV infections are endemic in most parts of the US [21, 22] with 80% of the infections remaining asymptomatic (CDC). This raises the concern that most people exposed to WNV are unaware of their seropositive status. We undertook this study with the objective to determine the role of pre-existing WNV antibodies in enhancing or inhibiting ZIKV infection in vitro. Serum samples were obtained either from healthy volunteers in the El Paso area that is endemic for WNV infections, or from subjects previously documented to have symptomatic WNV disease. A survey for WNV IgG antibodies via ELISA demonstrated a ~ 7% WNV sero-positivity rate in healthy volunteers. Further testing showed that WNV antibody positive sera significantly enhanced ZIKV infection in vitro with minimal neutralization. Interestingly, we did not find a difference between sera from WNV antibody positive symptomatic versus asymptomatic infections in terms of ZIKV ADE or neutralization. ZIKV ADE phenomenon was specific to WNV antibody positive sera as sera from ZIKV infected subjects showed effective ZIKV neutralization and ADE only at lower serum concentrations. We also found that WNV+ sera was more effective at neutralizing DENV-1 than ZIKV while the ADE phenomenon was not significantly different.

Collectively, our data demonstrates that WNV sero-positivity raises the possibility of enhancing ZIKV infection in individuals previously exposed to WNV infection. Whether these in vitro findings translate to an in vivo enhancement will need to be investigated in larger epidemiological studies from regions where both WNV and ZIKV are endemic.

## Results

### WNV sero-positivity and demographics of the study population

**Fig. 1:**
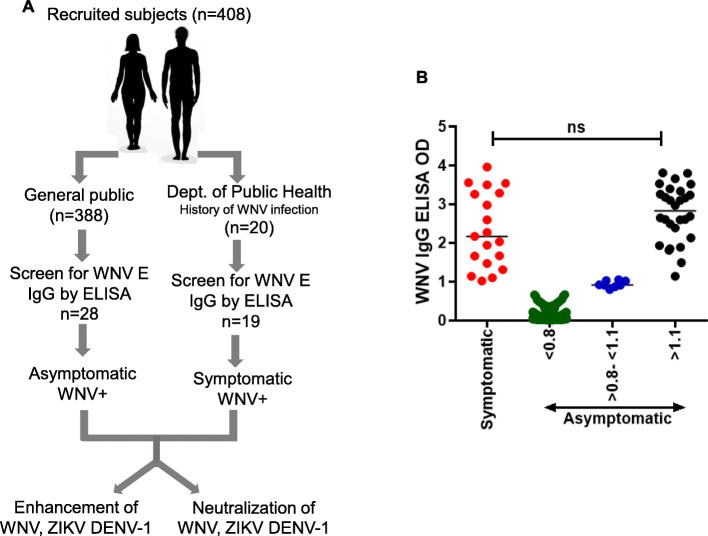
Schematic of subject recruitment and serum sample analysis for WNV E IgG antibodies. **a** Subjects were recruited either from the general public (asymptomatic) and self-reported to have no previous history of WNV infection or in collaboration with the Department of Public Health (symptomatic) and previously diagnosed with WNV infection. Samples from both groups of subjects were analyzed as indicated. **b** Analysis of WNV anti-E glycoprotein antibodies (IgG) in the serum samples obtained from 408 subjects via ELISA

The study involved recruiting two groups of subjects **1)** individuals from the general population in the El Paso area self-reported as never having been diagnosed with WNV infection (*N* = 388; asymptomatic) and **2**) subjects with a previous history of documented WNV infection recruited in collaboration with the public health department (*N* = 20; symptomatic). The study participants were requested to donate a blood sample and complete a study questionnaire. Serum samples from all the participants were analyzed for WNV Envelope IgG antibodies via ELISA (Fig. 1a). Of a total of 388 participants, 28 individuals (7.2%) were positive for WNV antibodies with an ELISA cut-off value of 1.1 or higher (Fig. 1b). Interestingly, 8 subjects showed borderline sero-positivity (ELISA OD > 0.8 to < 1.1) and could be due to the presence of low titer WNV antibodies or presence of cross reactive antibodies to related flaviviruses. Rest of the subjects showed serum ELISA OD value of 0.8 or lower. Samples were also obtained from individuals with a previous history of a symptomatic WNV infection and/or hospitalization in collaboration with the Public Health Department (City of El Paso). All of the symptomatic subjects showed ELISA O. D values of 1.1 or higher except one who was an OD value of 0.03; the reason for which remains undetermined **(**Fig. 1a**).** There was no significant difference (*p* = 0.09) between the WNV E IgG ELISA OD values of symptomatic versus asymptomatic individuals **(**Fig. 1b**).** Subject SMP003 was reported as having a symptomatic WNV infection to the public health department. However, neutralization and ADE data indicated that the subject was positive for ZIKV antibodies which was confirmed by ZIKV NS1 IgG ELISA (EUROIMMUN) (Table S1). Of the WNV seropositive samples, four (*N* = 4) were also positive for DENV1–4 NS1 IgG in an ELISA (Table S1 and S2).

The demographics of the study population are described in Tables 1 and 2**.** The healthy volunteer population comprised of 214 females (~ 55%) and 174 males (~ 45%) **(**Table 1**).** There was a significantly higher percentage (*p* = 0.0140) of subjects with age ≥ 50 in symptomatic WNV+ participants confirming that symptomatic WNV infections are often seen in older individuals **(**Table 2**).** There was a significantly higher percentage (*p* = 0.0166) of seropositive males than females **(**Table 3**)** which did not correlate with time spent outdoors (Table S3). Interestingly, certain zip codes of residence showed higher WNV sero-positivity for WNV (Fig. S1 and Table S4) which also correlated with occurrence of standing water (Table S5).

**Table 1:** Descriptive characteristics of baseline population

		No. (*n* = 388)	%
**Gender**	Male	174	44.8%
	Female	214	55.2%
**Age (years)**	< 50^a^	233	60.1%
	≥50	155	39.9%
**Race**	Hispanic	332	85.6%
	White	38	9.8%
	Black	5	1.3%
	Asian	6	1.5%
	Other^b^	7	1.8%

^a^ < 50: One respondent failed to answer age so, average age was input

^b^Other: includes mixed races and those who chose not to respond

**Table 2:** Comparison of descriptive characteristics of symptomatic and asymptomatic WNV+ subjects

	Symptomatic WNV+ subjects			Asymptomatic WNV+ subjects		
		No. (***n*** = 19^**a**^)	%	No.(***n*** = 28)	%	***p***-value
**Gender**	**Male**	11	57.9%	19	67.9%	0.5462
	**Female**	8	42.1%	9	32.1%	
**Age (years)**	**< 50**	3	15.8%	15	53.6%	0.0140
	**≥50**	16	84.2%	13	46.4%	
**Race**	**Hispanic**	10	52.6%	26	92.9%	ND
	**White**	8	42.1%	2	7.1%	
	**Black**	0	0.0%	0	0.0%	
	**Asian**	0	0.0%	0	0.0%	
	**Other**^**b**^	1	5.3%	0	0.0%	

^a^20 patients were referred from the public health department with a previous history of WNV diagnosis

^b^Other: includes mixed races and those who chose not to respond

*p*-value was determined using the Fisher exact test. ND-Not determined

However, only 19 tested positive in WNV E IgG ELISA

**Table 3:** WNV+ ELISA results segregated by sex

	Positive		Negative		***p***-value
	No.	%	No.	%	
**Male**	19	67.9%	155	43.2%	0.0166
**Female**	9	32.1%	205	56.8%	

P-value was determined using the Fisher exact test

### A high throughput assay for ADE using automated microscopy is comparable to flow cytometry based assay

**Fig. 2:**
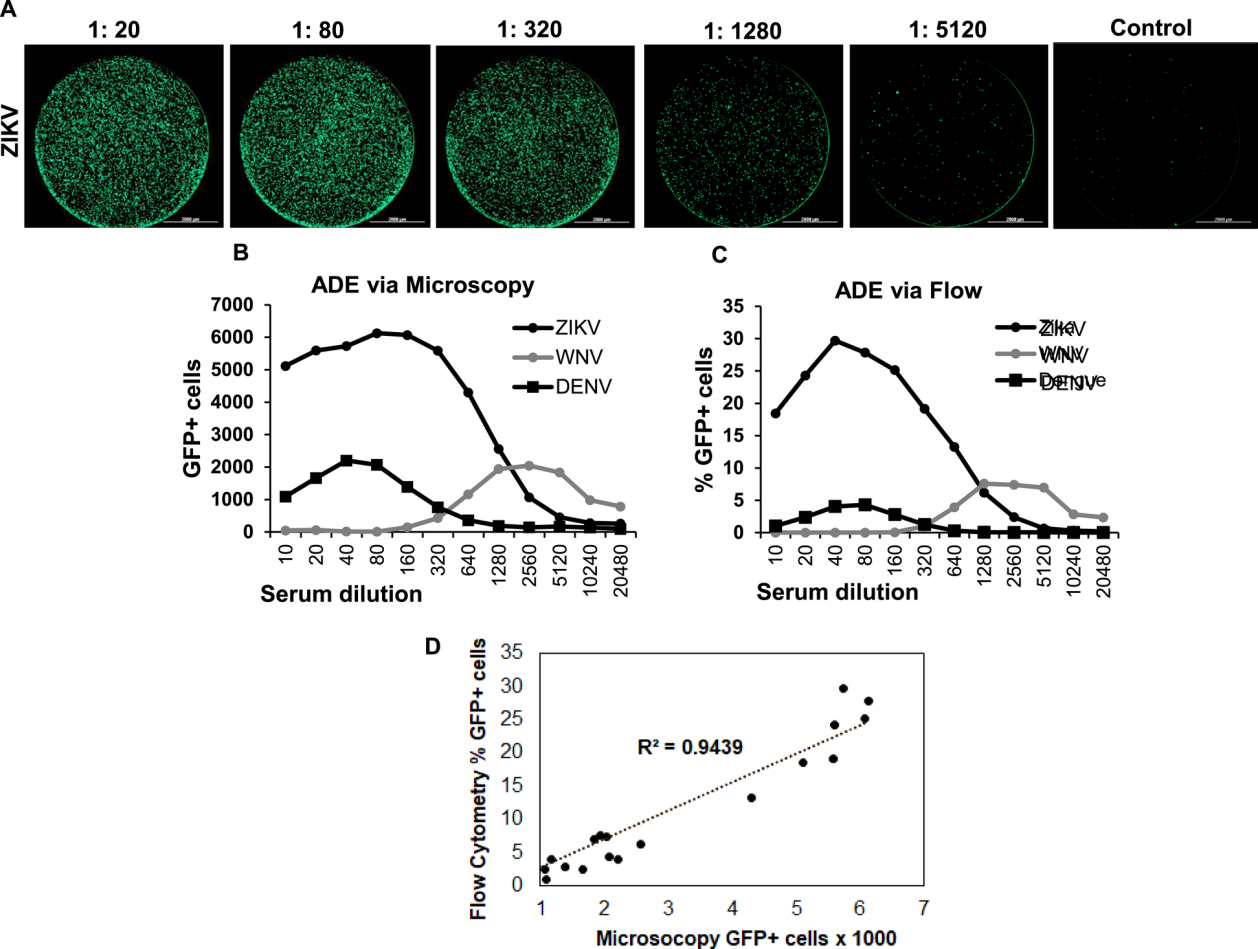
Automated microscopy based assay for ADE works comparable to flow cytometry. K562 cells were infected with ZIKV, WNV or DENV-1 RVPs in the presence of indicated dilutions of WNV immune sera from a symptomatic WNV+ subject. Samples were analyzed either by automated microscopy or via flow cytometry. **a** Representative microscopy images of whole wells of a 96 well plate infected with ZIKV RVPs in the presence of indicated dilutions of WNV immune sera. **b** Read out of enhancement of respective RVP infection by automated microscopy. **c** Read out of enhancement of respective RVP infection after flow cytometry analysis. **d** Correlation analysis of measurement of GFP+ cells by flow cytometry versus automated microscopy (R^2^ = 0.9439)

K562 cells bearing the Fc gamma receptor have been widely used to study ADE in vitro [23]. The cells are relatively non-permissive to flavivirus infection in the absence of specific binding antibodies. Binding of antibody to virus allows interaction of virus antibody complex with Fc gamma receptor on K562 cells that in turn permit virus uptake via endocytosis resulting in infection. While the K562 cell based ADE assay is widely used by researchers, the process involves infection of cells with live virus, staining cells for viral proteins as a measure of infection and quantifying the number of infected cells via flow cytometry. We wanted to develop a high throughput assay for ADE so larger number of samples could be tested against multiple viruses. Hence, we adapted the classical ADE assay in K562 cells using Reporter Virus Particles (RVPs) for infection (in 96 wells) and quantifying the number of infected cells via automated microscopy. We then compared the read out of ADE assay via automated microscopy and flow cytometry. K562 cells were infected with ZIKV, WNV or DENV-1 (Hawaii) RVPs in the presence of serial dilutions of WNV positive serum obtained from a symptomatic subject. Cells were analyzed 48 h post infection either by flow cytometry or automated microscopy. As demonstrated in Fig. 2**,** there was a concentration dependent enhancement of ZIKV **(**Fig. 2a**),** WNV and DENV RVP infection in the presence of specific sera. As expected, no infection was evident in the absence of enhancing sera (media control, Fig. 2a). Interestingly, results of the ADE assay via flow cytometry were similar to automated microscopy both with regards to peak enhancement as well as serum dilution resulting in maximum enhancement for each RVP **(**Fig. 2b & c**)**. Correlation analysis of measurement of GFP+ cells by flow cytometry versus automated microscopy was highly correlative with R^2^ = 0.9439 **(**Fig. 2d**).** Thus, the automated microscopy based assay can be readily used in a high throughput format to study the phenomenon of ADE.

### RVP based assay can differentiate between DENV, WNV and ZIKV sera in both ADE and neutralization assays

**Fig. 3:**
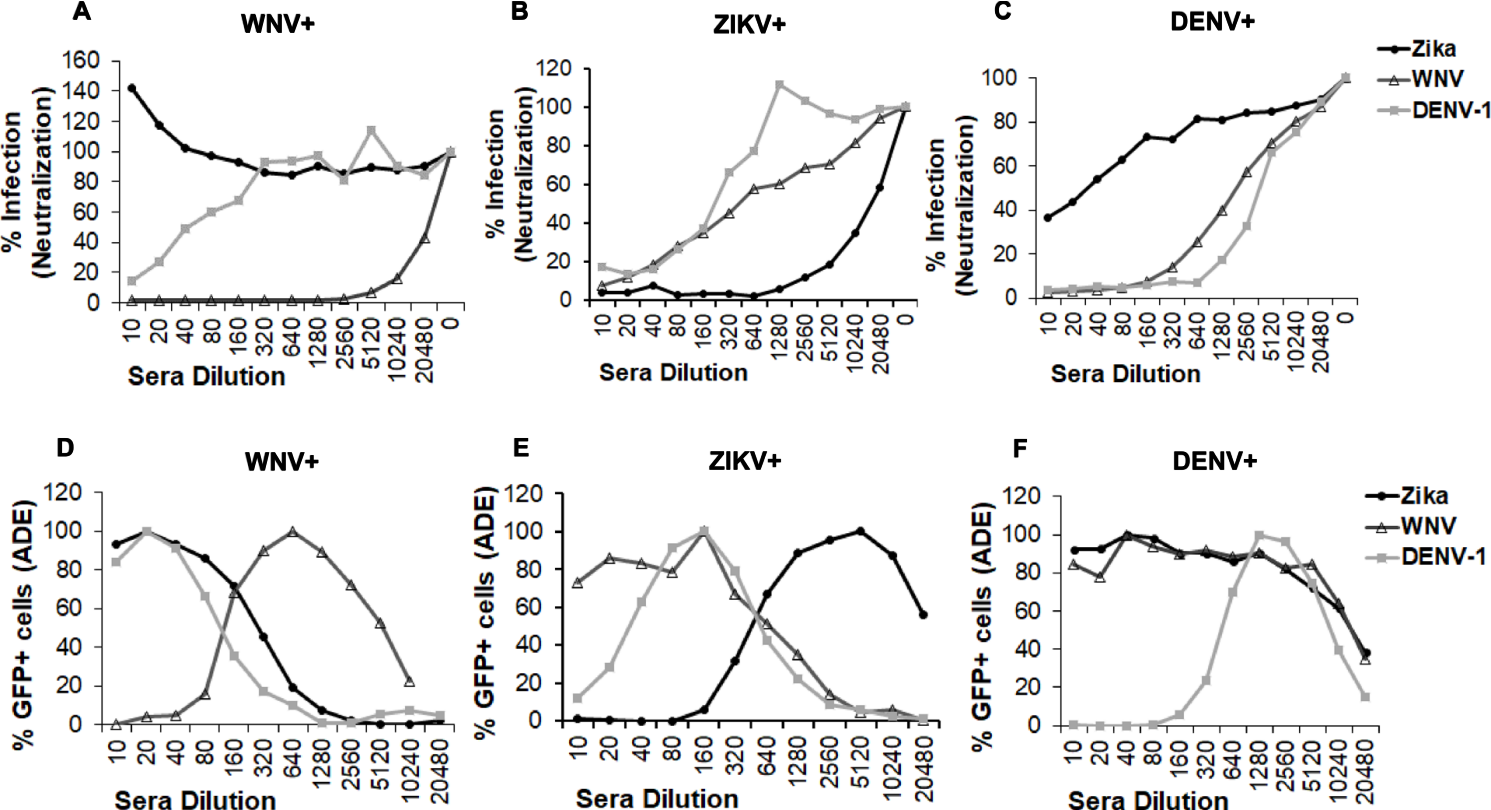
RVP based assay can differentiate between DENV, WNV and ZIKV sera in both ADE and neutralization assays. Vero cells were infected with (**a**) WNV (**b**) ZIKV or (**c**) DENV GFP RVPs in the presence of media alone or serial dilutions of WNV+, ZIKV+ or DENV+ sera samples. WNV+ sera was from a symptomatic WNV exposed subject as confirmed by WNV E IgG ELISA, and ZIKV and DENV+ sera were from subjects positive for ZIKV or DENV antibodies confirmed by specific NS1 IgG ELISA. The number of GFP positive cells was analyzed 72 h post infection. All wells were assayed in duplicates and graphs were plotted after normalizing data to 100% infection in the absence of any sera. K562 cells were infected with (**d**) WNV (**e**) ZIKV or (**f**) DENV GFP RVPs as in parts A-C above. The number of GFP positive cells was analyzed 48 h post infection. All wells were assayed in duplicates and graphs were plotted after normalizing data to peak ADE as 100% infection

We further validated the sensitivity of the high throughput RVP based assay to differentiate between antibodies elicited by WNV, DENV and ZIKV infection. Sera specific for each flavivirus was used in either a neutralization or ADE assay **(**Fig. 3**)** against the three different RVPs (WNV, ZIKV, DENV-1). Specifically, WNV+ sera was from a symptomatic WNV exposed subject as confirmed by WNV E IgG ELISA, and ZIKV and DENV+ sera were from subjects positive for ZIKV or DENV antibodies confirmed by specific NS1 IgG ELISAs. As demonstrated in Figs. 3 a-c**,** sera against each virus neutralized the specific virus most efficiently. Similarly, as evident in Figs. 3 d-f, ADE was seen at lower serum concentrations for the specific virus, while the cross-reactive RVPs showed ADE at higher serum concentrations. The clear differences in the IC_50_ for neutralization and the dilution of peak ADE provided a highly reliable assay to differentiate between these three closely related flaviviruses. The assay was highly reproducible as the error between wells was minimum and differences in peak ADE were higher than neutralization IC50s suggesting that an ADE assay might detect specific virus infection better than neutralization or when cross reactive neutralizing antibodies are present. Thus, in our hands, neutralization and ADE assays conducted simultaneously were capable of differentiating antibodies elicited by ZIKV, DENV and WNV infection.

### Sera samples from asymptomatic WNV seropositive subjects cause efficient WNV neutralization in vitro

**Fig. 4:**
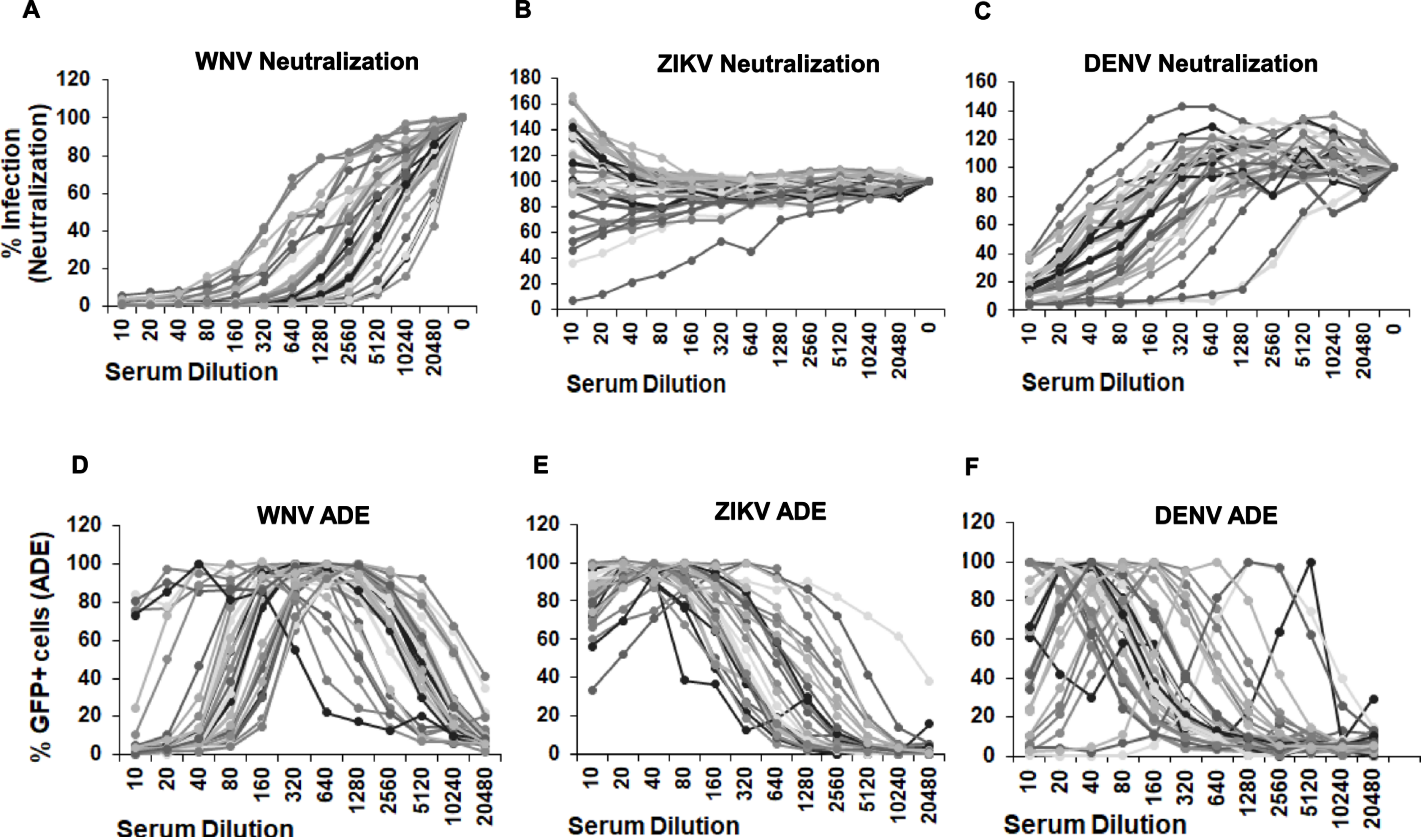
Sera samples from WNV seropositive donors cause enhancement of ZIKV infection. **a** Vero cells were infected with (**a**) WNV (**b**) ZIKV or (**c**) DENV RVPs in the presence of media alone or serial dilutions of WNV+ sera samples obtained from asymptomatic donors. The number of GFP positive cells was analyzed 72 h post infection as in Fig. 3 above. K562 cells were infected with (**d**) WNV, (**e**) ZIKV or (F) DENV RVPs in the presence of WNV+ sera samples from asymptomatic donors. The number of GFP positive cells was analyzed 48 h post infection

Having obtained an adequate sample size of WNV seropositive samples, we investigated the effect of WNV sero-positivity on neutralization and enhancement of WNV, ZIKV and DENV infection. As demonstrated in Fig. 4a**,** serum samples from asymptomatic WNV seropositive individuals effectively neutralized WNV infection even at lower serum concentrations. Some neutralization of DENV infection was also apparent at higher serum concentrations although not as effective as WNV neutralization **(**Fig. 4c**).** Surprisingly, there was minimum neutralization of ZIKV infection, with a general trend of no neutralization at all to enhancement of infection in some cases **(**Fig. 4b**).** A similar trend was seen for WNV, DENV and ZIKV neutralization from serum samples obtained from symptomatic WNV seropositive subjects (Fig. S2).

We next determined ADE of WNV, DENV and ZIKV infection in the presence of WNV+ sera. In accordance with the neutralization data, enhancement of WNV infection in the presence of WNV seropositive sera was observed only at lower serum concentrations with inhibition of infection seen at higher serum concentrations **(**Fig. 4d**).** However, with regards to ZIKV, for most samples significant enhancement of infection was seen at higher serum concentrations (1:10 dilution) with minimum neutralization **(**Fig. 4e**).** An intermediate phenomenon was seen with DENV with some inhibition apparent at higher serum concentrations followed by enhancement of infection at lower concentrations **(**Fig. 4f**).** A similar trend was seen for WNV, DENV and ZIKV ADE from serum samples obtained from symptomatic WNV seropositive subjects (Fig. S2). These data demonstrate that presence of WNV antibodies in the sera cause effective WNV neutralization but leads to ZIKV enhancement in in vitro assays. Moreover, there is individual variation evident in the phenotype of the sera in terms of neutralization and ADE that may be overlooked/minimized after pooling the data.

### Sera samples from WNV seropositive subjects lead to effective WNV neutralization but enhancement of ZIKV infection

**Fig. 5:**
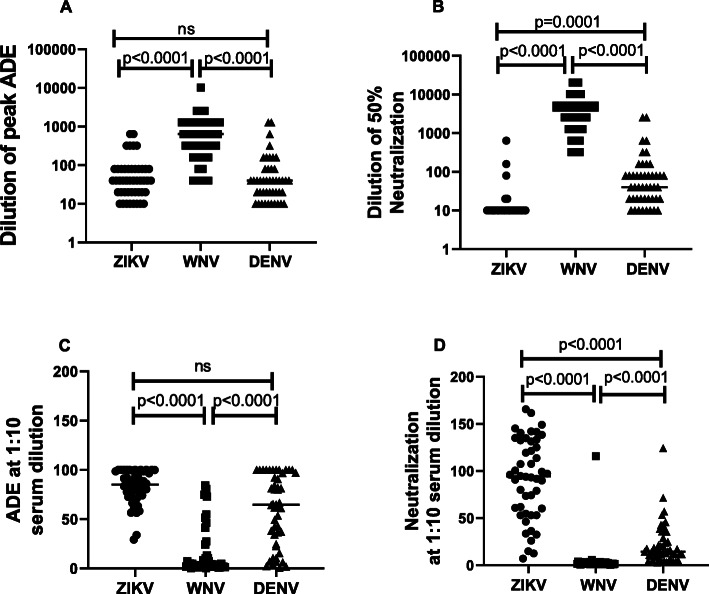
Sera samples from WNV seropositive subjects lead to effective WNV neutralization but enhancement of ZIKV infection. Serum samples from WNV seropositive donors (symptomatic and asymptomatic) were assayed for enhancement and neutralization of ZIKV, WNV and DENV RVPs. **a** Serum dilution that causes peak ADE for respective RVPs was determined. **b** Serum dilution that causes 50% neutralization of respective RVPs was determined. **c** ADE for ZIKV, WNV and DENV RVPs at a serum dilution of 1:10. **d** Neutralization of respective RVPs at a serum dilution of 1:10

We next analyzed pooled data from symptomatic and asymptomatic WNV seropositive subjects for neutralization and ADE of WNV, ZIKV and DENV. As demonstrated in Fig. 5a**,** the dilution of serum that caused peak ADE for WNV infection was significantly higher than dilution of serum that caused peak ADE for ZIKV infection. DENV ADE followed a similar trend as ZIKV ADE. When comparing neutralization, serum dilution that caused 50% neutralization of ZIKV was significantly lower than the serum dilution that led to 50% neutralization of WNV. Serum dilution for 50% DENV neutralization fell intermediate between ZIKV and WNV **(**Fig. 5b**).** A similar trend was seen when segregating the data based on symptomatic and asymptomatic WNV seropositive donors **(**Fig. S3) with no statistically significant difference between the groups. Along similar lines, we also compared pooled data for ADE and neutralization of infection at a serum dilution of 1:10, a relevant serum concentration in vivo*.* As demonstrated in Fig. 5c**,** there was a significant difference in ZIKV ADE when compared to WNV ADE at a 1:10 serum dilution. DENV ADE at 1:10 serum dilution was significantly higher than WNV but less than ZIKV ADE. When comparing neutralization at 1:10 serum dilution, both WNV and DENV infection was neutralized significantly better than ZIKV infection **(**Fig. 5d**).** Overall, these data revealed that pre-existing WNV antibodies while capable of neutralizing both WNV and DENV, poorly neutralize ZIKV infection. On the contrary, significant enhancement of ZIKV infection was evident even at higher serum concentrations.

### ZIKV antibody positive sera effectively neutralizes ZIKV with ADE seen at lower serum concentrations

**Fig. 6:**
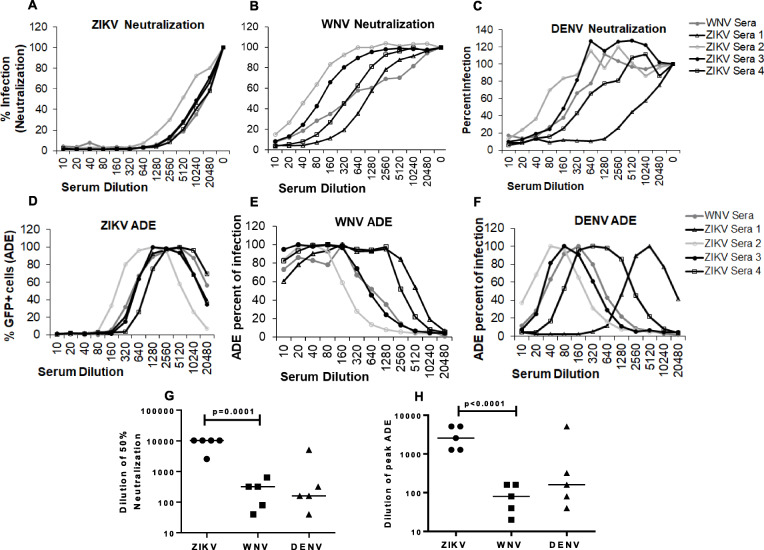
Sera from ZIKV seropositive donors effectively neutralizes ZIKV infection. Vero cells were infected with (**a**) ZIKV (**b**) WNV or (**c**) DENV GFP RVPs in the presence of media or serial dilutions of a WNV+ serum sample (symptomatic) or ZIKV+ serum samples obtained from BEI resources. The number of GFP positive cells was determined 72 h post infection. All wells were assayed in duplicates and percent infection determined after normalizing data to 100% infection in the absence of any sera. K562 cells were infected with (**d**) ZIKV (**e**) WNV or (F) DENV GFP RVPs in the presence of serial dilutions of a WNV+ serum sample (symptomatic) or ZIKV+ sera samples. The number of GFP positive cells was analyzed 48 h post infection and normalized to peak ADE as 100% infection. **g** Serum dilution that causes 50% neutralization of ZIKV, WNV and DENV RVPs was determined for each sample. **h** Serum dilution that causes peak ADE for WNV, ZIKV and DENV RVPs was determined

Our data above showed that WNV antibodies in the serum effectively neutralize WNV while enhancing ZIKV infection. We hence sought to investigate if the reverse was true and whether ZIKV+ sera would neutralize ZIKV and enhance WNV. To study this effect, we utilized serum samples from ZIKV+ human subjects **(**BEI Resources, Table S6) and analyzed their ability to neutralize or enhance ZIKV, WNV and DENV infection. As demonstrated in Fig. 6a**,** ZIKV+ sera effectively neutralized ZIKV infection. Moreover, peak ZIKV ADE was seen at lower serum concentrations with higher serum concentrations effectively inhibiting infection in K562 cells as well **(**Fig. 6d & h**)**. Interestingly, for WNV, neutralization was seen but at significantly higher serum concentrations than ZIKV (Fig. 6b & g**)**. With regards to WNV ADE, there was marked enhancement at higher serum concentrations with minimum neutralization **(**Fig. 6e and h**).** This phenomenon was similar to that seen in Fig. 5 regarding ZIKV enhancement in the presence of WNV serum **(**Fig. 6h**).** With regards to DENV neutralization, effective inhibition was evident at mean serum dilution of ~ 1:100 **(**Fig. 6 c & g**).** However, DENV ADE in the presence of ZIKV sera was not as pronounced with some inhibition of infection seen at higher serum concentrations and pronounced ADE at lower concentrations **(**Fig. 6 f and h**).** Taken together, these findings suggest that just like WNV+ sera enhances ZIKV, the reverse holds true for ZIKV+ sera. Furthermore, these data validate the sensitivity our RVP based assay for studying the ADE phenomenon.

### Neutralizing and non-neutralizing ZIKV antibodies behave similar to ZIKV+ serum with regards to ADE

**Fig. 7:**
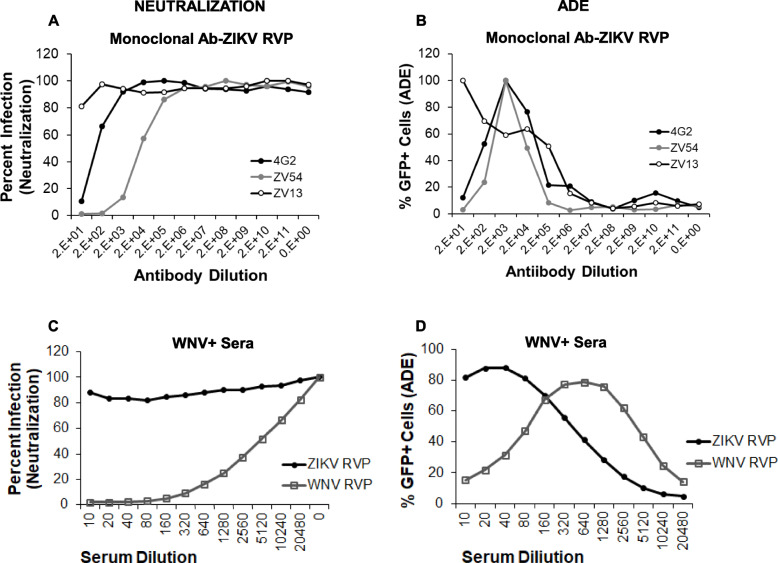
Non-neutralizing ZIKV antibodies cause enhancement of ZIKV infection at higher concentration without virus neutralization. **a** Vero cells were infected with ZIKV RVPs in the presence of different dilutions of ZIKV antibodies and number of GFP positive cells analyzed 72 h post infection. Percent infection was determined as above. **b** K562 cells were infected with ZIKV RVPs in the presence different dilutions of ZIKV antibodies and number of GFP positive cells analyzed 48 h post infection. Percent ADE was determined as above. **c** Pooled average of data from all WNV seropositive donors was plotted for neutralization of ZIKV and WNV infection. **d** Pooled average of data from all WNV seropositive donors was plotted for enhancement of ZIKV and WNV infection

The phenomenon of ADE is a complex process that remains poorly understood. In our studies above with human serum samples, enhancement of virus infection was apparent even when there was no neutralization of infection. To obtain further insights into the phenomenon, we utilized ZIKV neutralizing and non-neutralizing monoclonal antibodies in our RVP based ADE and neutralization assay. Two well characterized neutralizing antibodies (4G2- a pan-flaviviral mouse anti-dengue E protein and ZV-54, an anti-Zika E protein) and one non-neutralizing antibody (ZV-13, anti-Zika E protein) were used to understand how they behave in terms of enhancing and neutralizing ZIKV infection. As demonstrated in Fig. 7**a,** and as expected, the neutralizing antibodies 4G2 and ZV-54 effectively neutralized ZIKV infection while the non-neutralizing antibody ZV-13 did not. Interestingly, with regards to ZIKV enhancement, the neutralizing antibodies 4G2 and ZV-54 caused ADE at lower antibody concentrations, with higher concentrations actually inhibiting infection **(**Fig. 7b**).** However, the non-neutralizing antibody led to ZIKV enhancement even at higher concentrations with no inhibition of infection. The trend for neutralization and enhancement of ZIKV was strikingly similar to the pooled data for neutralization **(**Fig. 7c**)** and enhancement seen with WNV seropositive serum samples **(**Fig. 7d**).** Interestingly, our data shows different patterns of ADE curves for neutralizing vs non-neutralizing antibodies. Neutralizing antibodies show a peak ADE followed by decline whereas non- neutralizing antibodies form a plateau with a peak ADE reaching saturation. It is plausible that neutralizing antibodies cause enhancement at suboptimal concentrations unlike non-neutralizing antibodies. Coincidentally, the shape of the curves are reminiscent of what is seen with sera samples obtained from human subjects **(**Fig. 7c and d**).**

### Presence of cross-reactive glycoprotein E antibodies correlates with ZIKV ADE in vitro

**Fig. 8:**
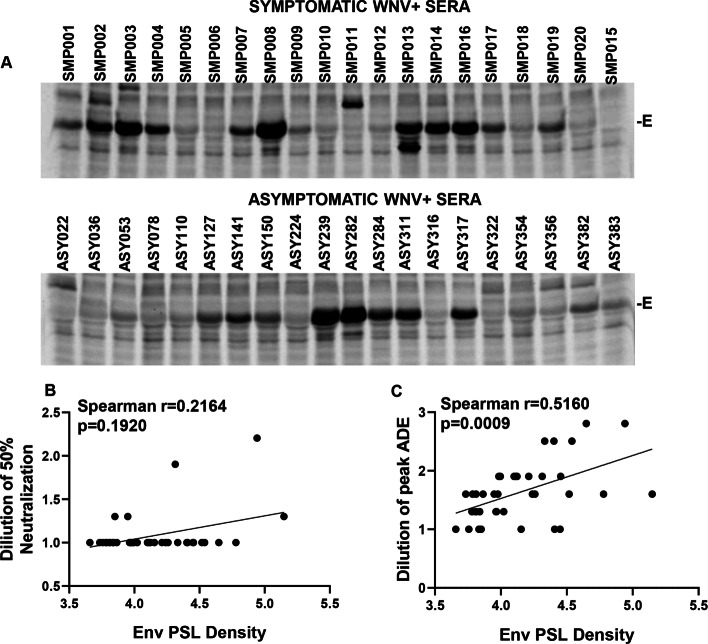
Prevalence of cross reactive E antibodies correlates with ZIKV neutralization and enhancement. **a** Stable cell line expressing Zika CprME-NS2B3 was radiolabeled with [^35^S]Met/Cys and cell lysates immunoprecipitated with Protein-A agarose beads coated with different WNV+ sera samples. Samples were resolved on and SDS-PAGE gel followed by PhosphorImager analysis. **b** Correlation analysis of Envelope band intensity with serum dilution causing 50% ZIKV neutralization. **c** Correlation analysis of Envelope band intensity with dilution of peak ZIKV ADE. Curves were fitted using GraphPad prism and simple linear regression. *P* values were obtained from two-tailed t test

Our studies with ZIKV monoclonal antibodies showed that non-neutralizing antibodies are potent inducers of ADE of ZIKV infection. Our results with the sera are also indicative of the presence of non- neutralizing antibodies against ZIKV in WNV+ sera. We hence asked whether we could detect anti-E antibodies against ZIKV in the WNV+ samples. Immunoprecipitation of cells lysates expressing the desired proteins provides a comprehensive overview of the repertoire of antibodies against different viral proteins in a sample. Studies have also implicated a role for antibodies other than the Envelope like prM in mediating ADE [24]. To study the cross-reactive antibody repertoire present in the WNV positive serum samples, we performed an immunoprecipitation (IP) analysis of ZIKV CprME expressing cell lysates with the indicated samples. As demonstrated in Fig. 8a and Fig. S4, the predominant antibodies present in the serum samples were against the E protein. We quantified the E protein bands in the IP gels and correlated it with neutralization and ADE of ZIKV. Interestingly, a higher intensity E protein band did not correlate with neutralization of ZIKV infection **(**Fig. 8b**)** but correlated significantly with ZIKV ADE **(**Fig. 8c**)** in vitro. These data confirm that the predominant antibodies responsible for enhancement of infection in case of ZIKV are directed towards the E protein and are most likely non-neutralizing antibodies.

## Discussion

Flaviviruses like WNV and ZIKV have not been a major human health concern due to the relatively mild and self-limiting disease [25]. However, in 2015, ZIKV attracted enormous attention from the scientific and healthcare community due to the sudden association of microcephaly with ZIKV infections worldwide [26]. On, the other hand, WNV infections are considered low priority due to the fact that majority of the infected individuals do not develop any symptoms. Individuals that do experience a symptomatic infection only develop a mild febrile illness [27]. The major concern of WNV infections is in less than 1% of the individuals, generally above 60 years of age, who develop severe disease with CNS symptoms such as encephalitis, meningitis etc. [28, 29]. Thus, more than 80% of the people exposed to WNV infections are unaware of their seropositive status.

As per CDC, West Nile is a nationally notifiable disease. WNV infections spread rapidly across the US after 1999 when the first cases were reported in Queens, NY [21, 22]. The infections have been reported in humans, birds and mosquitoes in 48 states and the District of Columbia [30, 31]. While WNV infections are considered endemic throughout the US, some regions in Nebraska, Wyoming, Montana, Colorado and the Dakotas have reported much higher seropositivity [21, 32, 33]. Consistent with the nationwide data, WNV infections are endemic in the state of Texas as well with several documented cases reported in El Paso each year.

The increase in the number of microcephaly cases during the Zika outbreaks in Brazil focused attention on the role of preexisting antibodies to related flaviviruses in exacerbating ZIKV disease. Several studies have been conducted on understanding the influence of existing DENV antibodies in enhancing or neutralizing ZIKV disease. Kam et al [34] demonstrated protection against ZIKV pathology in non-pregnant mice and lack of fetal retardation in pregnant mice in the presence of cross reactive DENV monoclonal antibody. Similarly, McCracken et al [35] and Pantoja et al [36] showed in separate studies that prior immunity to DENV did not alter parameters of ZIKV infection or pathogenesis in Rhesus Macaques despite evidence of ADE in cell culture based in-vitro assays. Swanstrom et al [37] showed neutralization of ZIKV infection by human antibodies isolated from DENV+ patients both in vitro and in mice model. On the contrary, a large number of studies, especially involving in vitro *tissue* culture models, have shown evidence of ZIKV enhancement in the presence of DENV specific antibodies in cells bearing the FcƴR [38–43]. This is similar to the well documented role of ADE in DENV infections, both in terms of natural infection and after DENV immunizations [18–20, 44]. ADE of DENV infection subsequent to a primary DENV exposure has been well documented in cell culture, mouse models, NHP and in humans [45].

While several studies have investigated the role of preexisting DENV antibodies in enhancing ZIKV infection, there have been limited studies addressing the influence of cross reactive WNV antibodies in ZIKV ADE. Interestingly, none of the previous studies investigated ZIKV ADE in context of antibodies elicited by symptomatic or asymptomatic WNV infection. Using sera samples from symptomatic or asymptomatic seropositive WNV subjects we found that there was no difference in the magnitude of ZIKV ADE in sera from these two groups. Interestingly, WNV+ sera was more potent at neutralizing DENV compared to ZIKV. Considering that WNV infections are seen throughout the US and that more than 80% of the infections are asymptomatic, these individuals could harbor ZIKV enhancing antibodies. These concerns would be even more relevant in the event of a future outbreak. Recently, Bardina et al [46] obtained plasma samples from convalescent WNV and DENV subjects and demonstrated enhancement of ZIKV infection in vitro in K562 cells. In the same study, passive transfer experiments in murine models showed a dose dependent effect of the immune plasma in modulating ZIKV infection. While higher concentration of passively transferred serum showed protection, lower concentrations showed enhancement of ZIKV infection.

In our study population, there was ~ 7% WNV sero-positivity in random samples obtained from a WNV endemic region of Texas by E IgG ELISA. The reactive samples were further tested for ZIKV NS1 IgG and DENV NS1 IgG to investigate the possibility of reactivity/exposure to a related flavivirus. One sample tested positive for ZIKV NS1 IgG and 4 tested positive for DENV1–4 NS1 IgG. This could be due to serological cross reactivity between the viruses [47] or dual exposure to the two viruses. Interestingly, while there was a slight but non-significant difference (*p* = 0.09) in the ELISA OD values between symptomatic and asymptomatic WNV infections, this did not translate to differences in ADE or neutralization between the groups. Overall, data from both symptomatic and asymptomatic WNV seropositive individuals demonstrated robust WNV neutralization with ADE seen at lower serum concentrations. On the other hand, with regards to ZIKV, WNV+ serum showed minimum neutralization even at higher serum concentrations with significant enhancement at the highest concentration of 1:10. Dengue RVPs were also neutralized although not as strongly as WNV but better than ZIKV. In this regard, a recent study showed that serum samples obtained from Mexican subjects with dengue hemorrhagic fever (DHF) were better at ZIKV neutralization and enhancement when compared to samples from subjects with dengue fever (DF) [48].

In our study, the ADE phenomenon was highly associated with the presence of flaviviral E antibodies. Immunoprecipitation data did not a reveal correlation with presence of antibodies to other structural proteins like prM. Studies with monoclonal antibody demonstrated that neutralizing antibodies showed peak shaped curves while non-neutralizing antibodies show plateau shaped curves in ADE assays. The enhancement with neutralizing antibodies is largely due to suboptimal levels of antibody at higher dilutions while non-neutralizing antibodies continue to enhance at lower dilutions as they lack neutralizing capacity. This difference in shape of ADE curves (plateau vs peaks) was also seen in ADE curves for ZIKV versus WNV using the sera samples. However, we must emphasize that the ADE phenomenon did vary between individuals with a minority of WNV+ subjects showing some neutralization of ZIKV. Collectively, our findings suggest that WNV+ sera has excess of neutralizing antibodies against WNV but largely non-neutralizing antibodies against ZIKV. Our RVP assay was found to be a convenient high throughput assay that can be used to study a large number of serum samples against multiple flaviviruses simultaneously.

Our study is the first to analyze samples from symptomatic and asymptomatic WNV seropositive individuals and understand their ability to neutralize or enhance ZIKV, WNV and DENV infection simultaneously. These findings may have implications for WNV endemic regions with ZIKV co-circulation, especially in women of childbearing age.

## Conclusions

ADE of virus infections is a common observation especially seen when antibody concentrations decline or are derived from a heterologous virus unable to neutralize the specific infection. In this study, we tried to understand if presence of WNV specific antibodies derived from human subjects enhance or inhibit ZIKV infection in cell culture. Our study shows that sera derived from WNV antibody positive subjects significantly enhances ZIKV infection in vitro with minimum neutralization. This has important implications, especially for women of childbearing age as WNV infections are endemic in the US. As most of these infections are asymptomatic, people are unaware of their seropositive status. Our study also sets precedent for further evaluations in larger cohorts and in geographical regions where ZIKV and WNV infections co-circulate.

## Methods

### Ethics, consent and permissions

The study was reviewed and approved by the Texas Tech University Health Sciences Center’s regional Institutional Review Board (IRB). All methods were performed in accordance with the relevant guidelines and regulations. The study design was cross-sectional and the study number recorded as IRB# E17056, approval date 03/16/2017. All participants were provided with written and oral information about the study. Written informed consent of all study participants in accordance with the Institutional policy was documented. All participants were identified by coded numbers to assure anonymity and all patient records kept confidential.

### Study population, sample collection and storage

Three hundred and eighty-eight (*N* = 388) healthy human volunteers were recruited from a WNV endemic region of Texas. Twenty subjects (*N* = 20) with a previous history of symptomatic WNV infection were also recruited in collaboration with the Department of Public Health, City of El Paso, Texas. As WNV is a notifiable disease, symptomatic patients in the local hospitals reported to the public health department were confirmed for WNV infection by testing for presence of specific IgM antibodies in the serum or cerebrospinal fluid (CSF). Officials from the public health department were requested to contact these subjects to participate in the study. The average duration of time lapse from the date of WNV diagnosis to sample collection was ~ 2.2 years. To determine appropriate sample size for the study, statistical analysis was conducted to calculate required sample size for a sero-prevalence study with a 95% confidence interval. We anticipated the sero-prevalence rate for WNV antibodies in our population to vary from 3 to 10%. Considering those estimates, a sample size of > 217 would have adequate power. Each subject was requested to complete a questionnaire and provide a blood sample in a serum separator tube and the serum was aliquoted and stored at − 70 °C until further analysis. The questionnaire was administered to obtain demographic data and other information pertinent to the study.

### Cells and reagents

Experiments were conducted in BSL2 containment following appropriate safely protocols and guidelines. 293 T and Vero cells were obtained from American Type Culture Collection (ATCC) and cultured in Dulbecco’s Modified Eagle’s Medium (DMEM) supplemented with 10% Fetal Bovine Serum (FBS). K562 cells were obtained from ATCC and cultured in Roswell Park Memorial Institute (RPMI) medium supplemented with 10% FBS. Transfections were performed using Turbofect reagent (Thermo Fisher) as per the manufacturer’s instructions. The WNV plasmid containing the sub-genomic GFP expressing replicon derived from lineage II strain of WNV-Rep/GFP, WNV CprME and Zika CprME expression vectors have been described previously [49, 50]. The DENV-1(Hawaii) virus was obtained from the Biodefense and Emerging Infections Research Resources Repository (BEI resources). Viral RNA was used to amplify the CprME region using specific primers and RT-PCR followed by cloning into pCDNA3.1 TOPO vector (Invitrogen). The ZIKV immune sera from seropositive individuals was kindly provided by BEI resources. The ZIKV neutralizing and non-neutralizing antibodies 4G2 (neutralizing), ZV-13 (non-neutralizing) and ZV-54 (neutralizing), were obtained from Millipore Sigma (St. Louis, MO). The 4G2 antibody binds to the fusion loop at the extremity of domain II of E protein of different flaviviruses. The ZV-54 antibody binds to the DIII of the envelope protein corresponding to the lateral ridge and the ZV-13 binds to the conserved FL in DII.

### Elisa

The WNV E IgG ELISA, DENV NS1 Type 1–4 IgG ELISA and ZIKV NS1 IgG ELISA kits were obtained from EUROIMMUN (Luebeck, Germany) and used as per the manufacturer’s protocol. The anti-WNV ELISA (IgG) is intended for the detection of IgG antibodies to WNV in human serum and plasma samples. The antigen source is a recombinant detergent extracted glycoprotein E of WNV from the membrane fraction of human cells. The anti-WNV antibodies bind to the antigen coated in microtiter wells followed by addition of anti-human IgG HRP conjugate that results in a calorimetric reaction on addition of TMB substrate. The controls and calibrator included on the kit must be used in each run and results are evaluated by calculating a ratio of OD value of patient sample over OD value of the calibrator. A ratio of ≥1.1 is considered as positive for WNV IgG antibodies.

### Generation and titration of reporter virus particles (RVPs)

RVPs were prepared by transfecting 293 T cells with WNV Rep/GFP replicon construct along with WNV or ZIKV or DENV-1 CprME expression constructs. The RVPs were harvested 48 h post transfection, aliquoted and stored for future use. RVPs were titrated in Vero cells plated in 96 well clear bottom black plates at 5000 cells per well. Serial two-fold dilutions of the RVPs were used to infect Vero cells and incubated for 72 h. The plates were fixed using 4% formalin in PBS and images acquired using the Cytation5 imaging system (BioTek, Winooski, VT, USA). Images of whole wells were acquired and the number of GFP+ cells per well was quantified using Gen5 software (BioTek).

### Neutralization and ADE assays

For neutralization assays, human sera or antibodies were serially diluted in appropriate media (DMEM or RPMI) and incubated with the RVPs for 1 h at 37 °C. For control wells, an equivalent volume of media was added. Sera samples were heat inactivated at 56 °C for 30 min prior to dilution. Subsequently, the virus:sera mix was added to Vero cells and number of GFP positive cells quantitated 72 h post infection as described above. Percent neutralization was calculated after normalizing data to cells infected in the absence of any sera (media control). Assays for ADE were conducted as described above except FcƴRII bearing K562 cells were used instead of Vero and plates read 48 h post infection. ADE data was normalized to the wells that showed maximum enhancement as detected by number of GFP+ cells.

### Immunoprecipitation

The protocol for radiolabeling and immunoprecipitation of cell and virus lysates has been described previously [51]. Briefly, the stable cell line Zika-CprME-NS2B3-E2 [52] was washed with RPMI medium lacking Methionine and Cysteine. Thereafter, cells were incubated in RPMI medium supplemented with FBS and [^35^S]Met/Cys. Cells were lysed with Triton X containing lysis buffer and cell lysates immunoprecipitated with Protein-A beads coated with WNV+ human serum samples. Immunoprecipitated lysates were washed, resolved by SDS-PAGE followed by PhosphorImager analysis.

### Statistical analysis

Statistical analysis was conducted using GraphPad Prism (GraphPad Software, San Diego, CA). Comparison between multiple groups was done using the Kruskal-Wallis test. Spearman’s correlation with linear regression was used for all correlation determination using the GraphPad Prism Software. *P* values were considered significant at *p* < 0.05.

## Supplementary Information

**Additional file 1: Supplementary Figures.** Figures S1-S4.

**Additional file 2:** **Supplementary Tables.** Tables S1-S6.

## Data Availability

The datasets used and/or analyzed during the current study are available from the corresponding author on reasonable request.

## Abbreviations

ZIKV: Zika virus
WNV: West Nile Virus
DENV: Dengue Virus
ADE: Antibody Dependent Enhancement
PHEIC: Public Health Emergency of International Concern
CZS: Congenital Zika Syndrome
RVP: Reporter Virus Particles
