# Supplementary material for: Enhancement of Zika virus infection by antibodies from West Nile virus seropositive individuals with no history of clinical infection
Source: BMC Immunol. 2021 Jan 9;22:5. doi: 10.1186/s12865-020-00389-2 (PMC7796652; doi:10.1186/s12865-020-00389-2)
Supplement: Supplementary file 1 — Additional file 1: Supplementary Figures. Figures S1-S4. [file 12865_2020_389_MOESM1_ESM.pptx]

## Slide 1
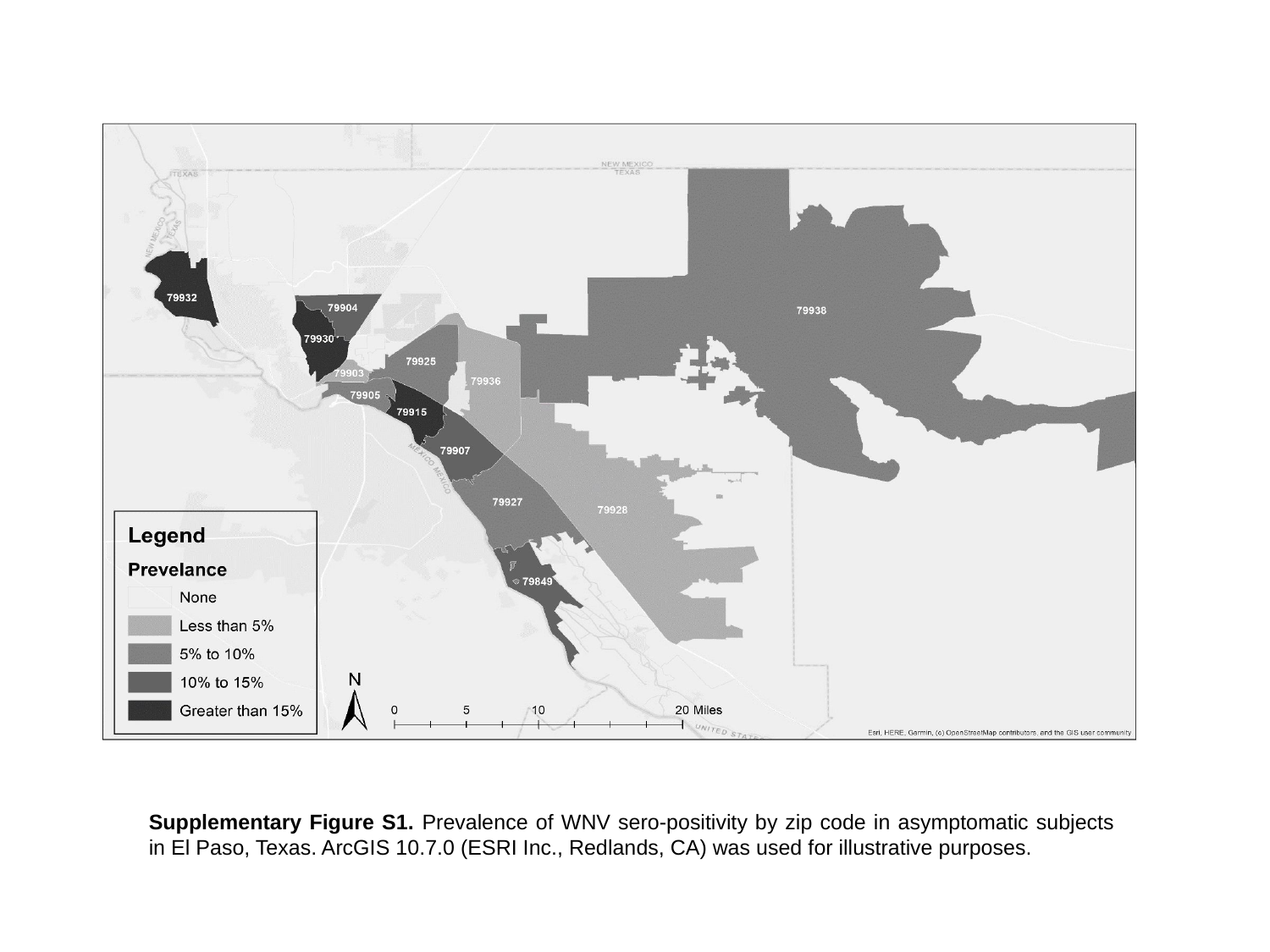

Supplementary Figure S1. Prevalence of WNV sero-positivity by zip code in asymptomatic subjects in El Paso, Texas. ArcGIS 10.7.0 (ESRI Inc., Redlands, CA) was used for illustrative purposes.

## Slide 2
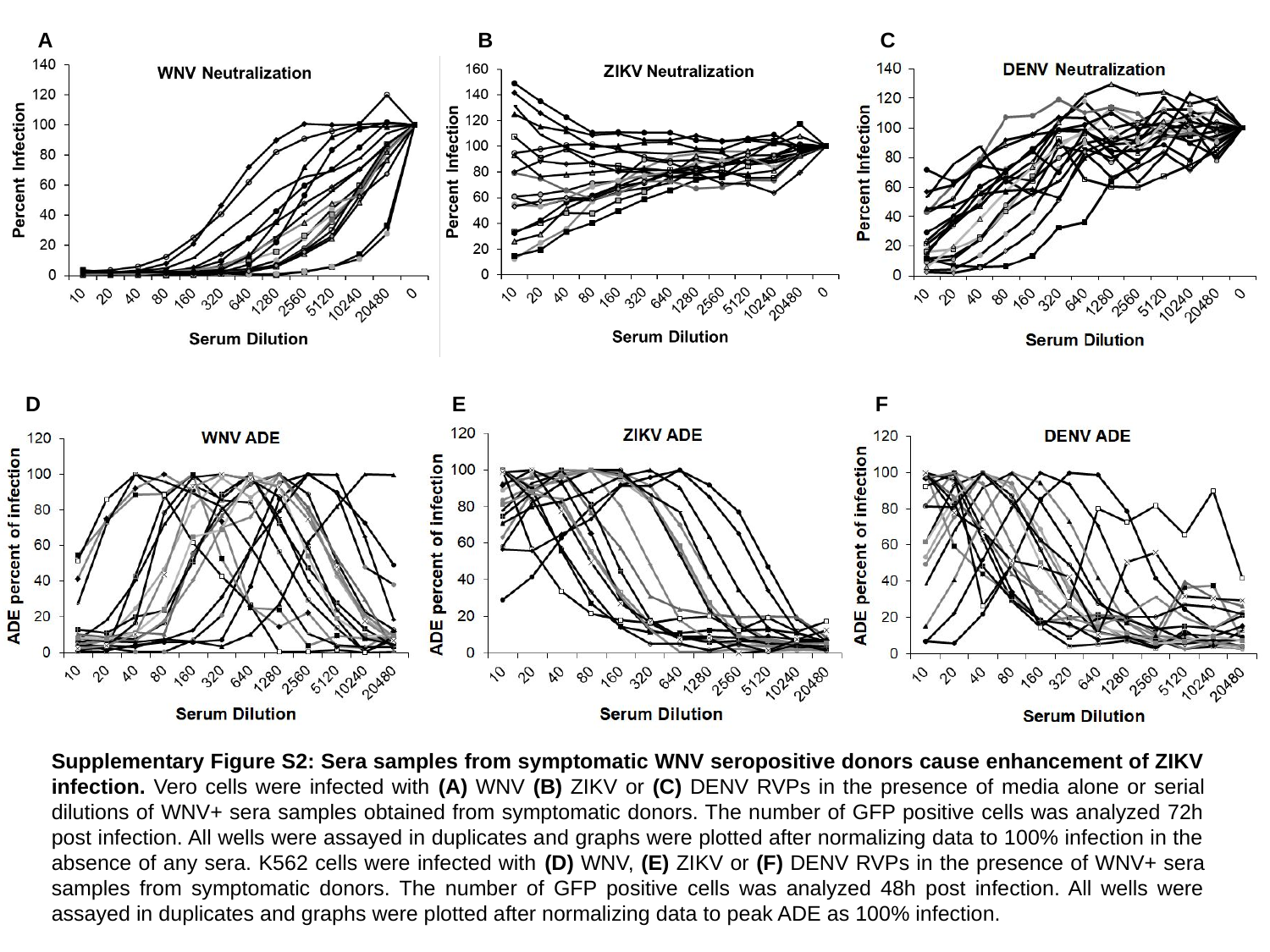

C
A
B
F
E
D
Supplementary Figure S2: Sera samples from symptomatic WNV seropositive donors cause enhancement of ZIKV infection. Vero cells were infected with (A) WNV (B) ZIKV or (C) DENV RVPs in the presence of media alone or serial dilutions of WNV+ sera samples obtained from symptomatic donors. The number of GFP positive cells was analyzed 72h post infection. All wells were assayed in duplicates and graphs were plotted after normalizing data to 100% infection in the absence of any sera. K562 cells were infected with (D) WNV, (E) ZIKV or (F) DENV RVPs in the presence of WNV+ sera samples from symptomatic donors. The number of GFP positive cells was analyzed 48h post infection. All wells were assayed in duplicates and graphs were plotted after normalizing data to peak ADE as 100% infection.

## Slide 3
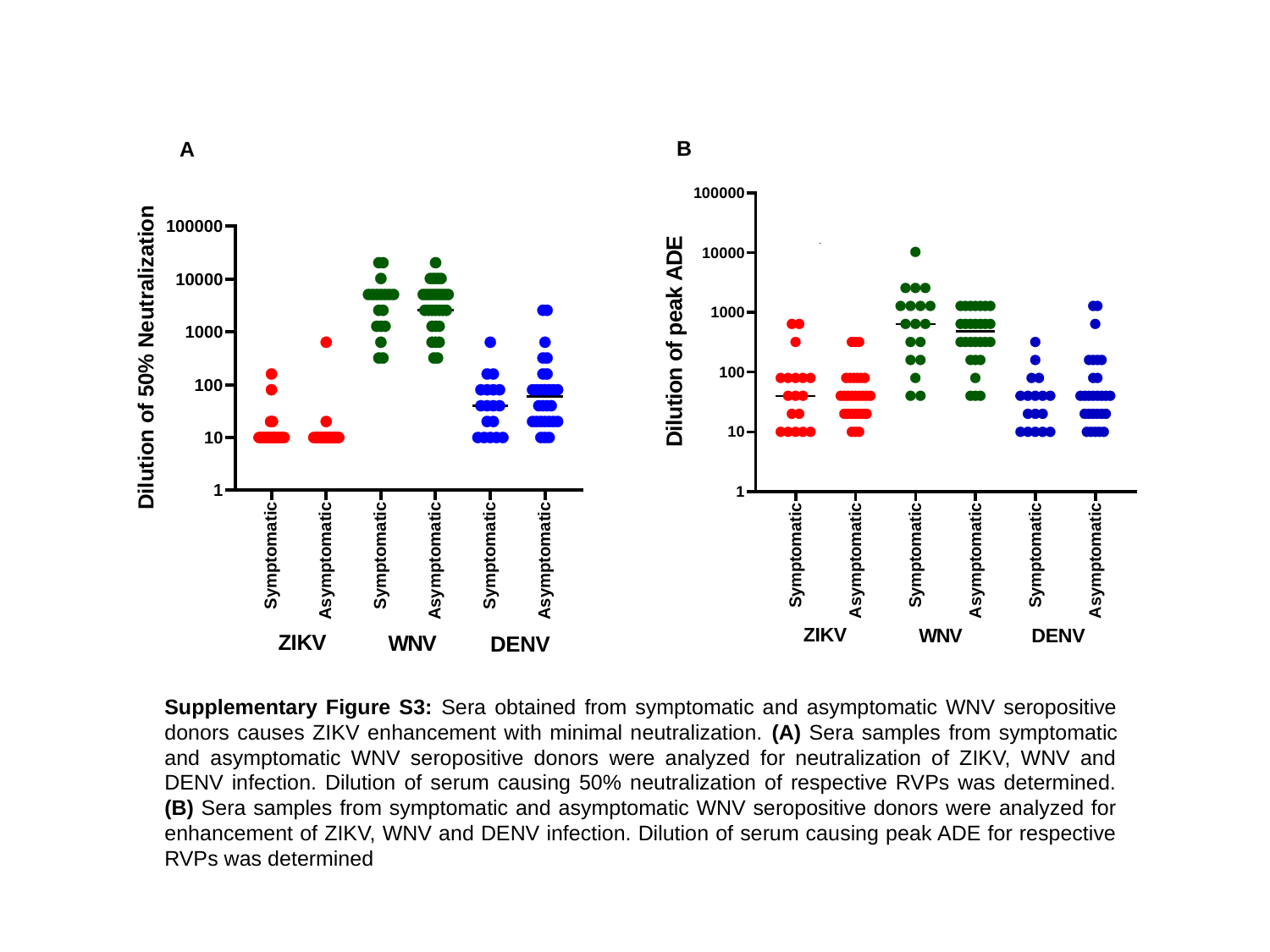

B
A
Supplementary Figure S3: Sera obtained from symptomatic and asymptomatic WNV seropositive donors causes ZIKV enhancement with minimal neutralization. (A) Sera samples from symptomatic and asymptomatic WNV seropositive donors were analyzed for neutralization of ZIKV, WNV and DENV infection. Dilution of serum causing 50% neutralization of respective RVPs was determined. (B) Sera samples from symptomatic and asymptomatic WNV seropositive donors were analyzed for enhancement of ZIKV, WNV and DENV infection. Dilution of serum causing peak ADE for respective RVPs was determined

## Slide 4
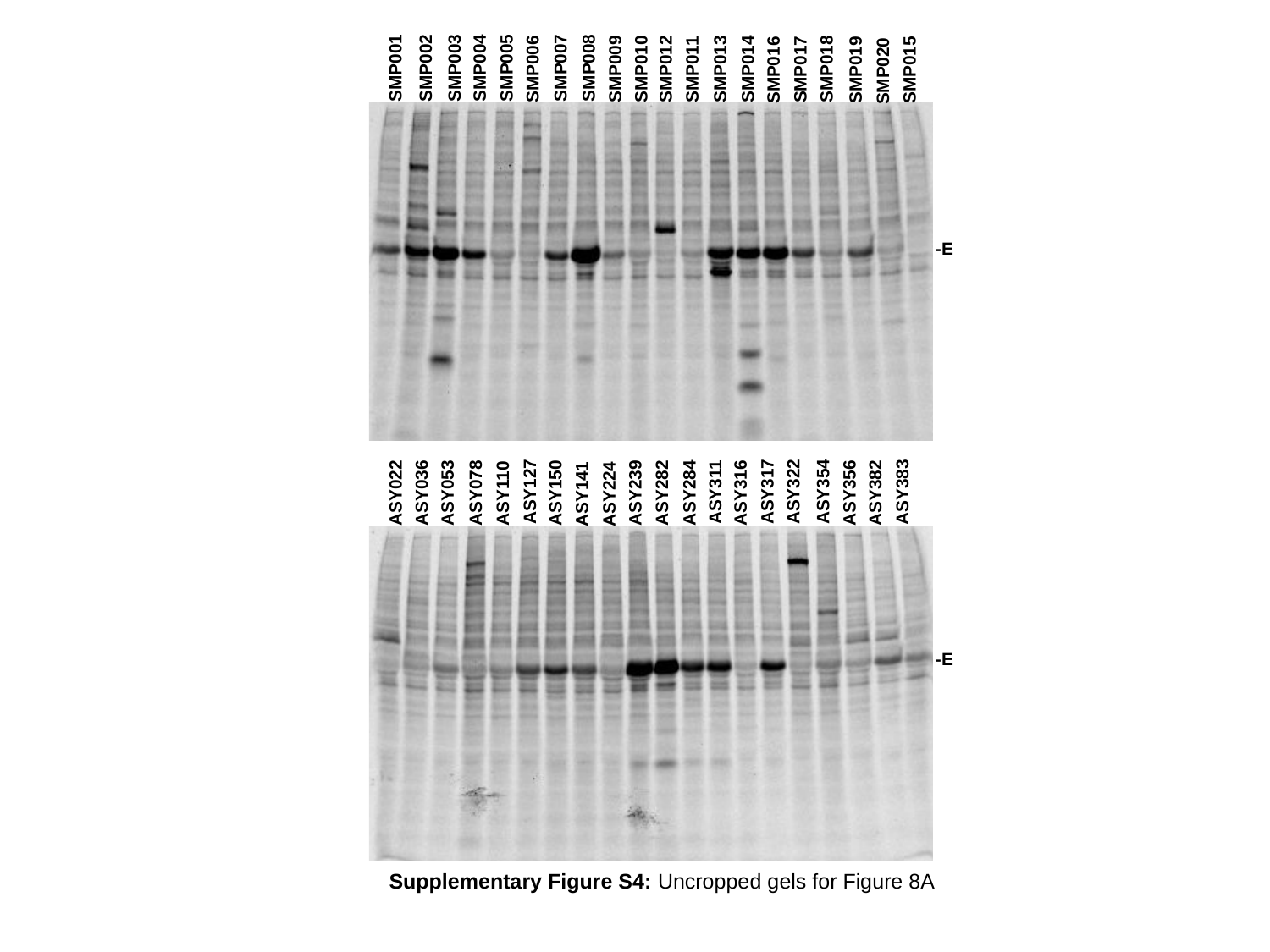

SMP003
SMP004
SMP005
SMP007
SMP008
SMP001
SMP002
SMP018
SMP013
SMP014
SMP012
SMP011
SMP006
SMP009
SMP010
SMP017
SMP016
SMP019
SMP015
SMP020
-E
ASY317
ASY322
ASY354
ASY311
ASY127
ASY383
ASY382
ASY356
ASY284
ASY316
ASY078
ASY110
ASY150
ASY022
ASY036
ASY053
ASY282
ASY239
ASY141
ASY224
-E
Supplementary Figure S4: Uncropped gels for Figure 8A
