# Supplementary material for: Enhancement of Zika virus infection by antibodies from West Nile virus seropositive individuals with no history of clinical infection
Source: BMC Immunol. 2021 Jan 9;22:5. doi: 10.1186/s12865-020-00389-2 (PMC7796652; doi:10.1186/s12865-020-00389-2)
Supplement: Supplementary file 2 — Additional file 2: Supplementary Tables. Tables S1-S6. [file 12865_2020_389_MOESM2_ESM.docx]

| **Table S1:** Reactivity of symptomatic WNV+ samples in ZIKV and DENV1 NS1 IgG ELISA | | | |
| --- | --- | --- | --- |
| **Sample** | **WNV E IgG** | **ZIKV NS1 IgG** | **DENV NS1 IgG** |
| **SMP 001** | **3.56** | **0.16** | **0.33** |
| **SMP 002** | **2.60** | **0.22** | **0.27** |
| **SMP 003** | **1.31** | **3.14** | **0.96** |
| **SMP 004** | **3.29** | **0.14** | **0.27** |
| **SMP 005** | **2.03** | **0.04** | **0.19** |
| **SMP 006** | **1.14** | **0.09** | **0.16** |
| **SMP 007** | **3.50** | **0.13** | **0.81** |
| **SMP 008** | **3.96** | **0.11** | **0.72** |
| **SMP 009** | **1.94** | **0.08** | **0.11** |
| **SMP 010** | **2.17** | **0.12** | **0.86** |
| **SMP 011** | **1.02** | **0.09** | **0.06** |
| **SMP 012** | **1.10** | **0.06** | **0.1** |
| **SMP 013** | **2.98** | **0.1** | **0.42** |
| **SMP 014** | **3.54** | **0.27** | **1.25** |
| **SMP 015** | **0.03** | **0.05** | **0.1** |
| **SMP 016** | **3.26** | **0.1** | **0.84** |
| **SMP 017** | **2.27** | **0.18** | **0.39** |
| **SMP 018** | **1.67** | **0.14** | **0.56** |
| **SMP 019** | **1.66** | **0.07** | **0.44** |
| **SMP 020** | **1.47** | **0.13** | **0.87** |

ELISA OD >1.1=Positive.

ZIKV and DENV positive samples marked in red.

| **Table S2:** Reactivity of asymptomatic WNV+ samples in ZIKV and DENV1 NS1 IgG ELISA | | | |
| --- | --- | --- | --- |
|  | **WNV E IgG** | **ZIKV NS1 IgG** | **DENV NS1 IgG** |
| **ASY 022** | **2.49** | **0.14** | **0.38** |
| **ASY 036** | **3.10** | **0.09** | **0.21** |
| **ASY 053** | **3.40** | **0.14** | **0.45** |
| **ASY 078** | **3.26** | **0.24** | **0.56** |
| **ASY 110** | **2.39** | **0.22** | **0.44** |
| **ASY 127** | **2.61** | **0.28** | **0.63** |
| **ASY 141** | **1.81** | **0.15** | **0.39** |
| **ASY 150** | **3.16** | **0.08** | **0.18** |
| **ASY 224** | **3.34** | **0.3** | **0.85** |
| **ASY 239** | **3.66** | **0.11** | **0.56** |
| **ASY 280** | **2.60** | **0.16** | **0.38** |
| **ASY 282** | **2.61** | **0.49** | **2.66** |
| **ASY 284** | **3.80** | **0.08** | **0.46** |
| **ASY 291** | **1.14** | **0.19** | **0.68** |
| **ASY 294** | **1.49** | **0.44** | **3.01** |
| **ASY 301** | **2.65** | **0.05** | **0.19** |
| **ASY 302** | **3.18** | **0.16** | **0.47** |
| **ASY 304** | **3.52** | **0.06** | **0.47** |
| **ASY 308** | **3.10** | **0.13** | **0.83** |
| **ASY 311** | **3.81** | **0.31** | **1.18** |
| **ASY 316** | **1.85** | **0.07** | **0.16** |
| **ASY 317** | **1.89** | **0.16** | **0.55** |
| **ASY 322** | **1.93** | **0.03** | **0.11** |
| **ASY 356** | **3.24** | **0.05** | **0.35** |
| **ASY 361** | **3.53** | **0.04** | **0.38** |
| **ASY 378** | **2.97** | **0.17** | **0.66** |
| **ASY 382** | **2.69** | **0.14** | **0.75** |
| **ASY 383** | **2.13** | **0.1** | **0.36** |

ELISA OD >1.1=Positive.

ZIKV and DENV positive samples marked in red.

| **Table S3: Relationship of asymptomatic WNV seropositive males and females with outdoor activity** | | | | |
| --- | --- | --- | --- | --- |
| **Outdoor Activity** | **YES** | **NO** | **% YES** | **p-value** |
| **Male** | 13 | 6 | 68% | 0.114 |
| **Female** | 3 | 6 | 33% |  |

P-value was determined using the Fisher exact test.

| **Table S4: WNV IgG sero-positivity Prevalence based on Zip Codes** | | | |
| --- | --- | --- | --- |
| **Zip Code** | **Cases** | **Total** | **Prevalence (%)** |
| 79849 | 1 | 8 | 12.5 |
| 79903 | 1 | 21 | 4.76 |
| 79904 | 2 | 17 | 11.76 |
| 79905 | 2 | 24 | 8.33 |
| 79907 | 3 | 27 | 11.11 |
| 79915 | 6 | 38 | 15.78 |
| 79925 | 1 | 15 | 6.66 |
| 79927 | 3 | 31 | 9.67 |
| 79928 | 1 | 25 | 4 |
| 79930 | 3 | 19 | 15.78 |
| 79932 | 1 | 6 | 16.66 |
| 79936 | 1 | 44 | 2.27 |
| 79938 | 2 | 28 | 7.14 |

Positive case in Zip Code 32500 was not included in this data as it is not in the El Paso Area.

Lowest Prevalence Zip Codes = 79903, 79925, 79928, 79936, 79938

High Prevalence Zip Codes = 79849, 79904, 79905, 79907, 79915, 79927, 79930, 79932.

| **Table S5. Standing Water in High Prevalence WNV+ Zip Codes (n = 170)** | | | | | |
| --- | --- | --- | --- | --- | --- |
|  | | **Positive** | | **Negative** | |
|  |  | **No.** | **%** | **No.** | **%** |
| **Standing Water** | **Yes** | 11 | 52.4% | 61 | 40.9% |
|  | **No** | 10 | 47.6% | 88 | 59.1% |

Prevalence Odds Ratio =1.59 (Out of the high prevalence Zip codes, The proportion of people with a positive WNV ELISA test is ~1.59 fold greater if the person lives near standing water).

| **Table S6. ZIKV+ serum samples used in the study** | | | |
| --- | --- | --- | --- |
| **Study No.** | **BEI-Batch No.** | **BEI Item No.** | **Description** |
| **ZKIV Sera 1** | 202215924 | NR-51007 | Convalescent Huma serum from ZIKV+ patient, 112 days post onset of symptoms |
| **ZKIV Sera 2** | 202691631 | NR-50985 | Convalescent Huma serum from ZIKV+ patient, 178 days post onset of symptoms |
| **ZKIV Sera 3** | 202376492 | NR-51013 | Convalescent Huma serum from ZIKV+ patient, 183 days post onset of symptoms |
| **ZKIV Sera 4** | 202802653 | NR-51021 | Convalescent Huma serum from ZIKV+ patient, 186 days post onset of symptoms |
